# Efficient macrocyclization facilitated by skeleton preorganization†

**DOI:** 10.1039/d3ra05671f

**Published:** 2023-10-24

**Authors:** Rong-Yao Xu, Xiu Liu, Guang Sun, Zhi-Yuan Zhang, Ming Dong, Liya Zhao, Si-Miao Zhang, Xi-Yang Wang, Hong-Xu Zhang, Shang-Jie Yang, Xiuguang Wang, Bin Li, Jian Li, Chunju Li

**Affiliations:** a Tianjin Key Laboratory of Structure and Performance for Functional Molecules, College of Chemistry, Tianjin Normal University Tianjin 300387 P. R. China zzy@tjnu.edu.cn hxxydongm@tjnu.edu.cn cjli@tjnu.edu.cn; b School of Chemistry and Chemical Engineering, Henan Normal University P. R. China

## Abstract

Reported here is the efficient macrocyclization facilitated by skeleton preorganization. A pyridylcarbazole macrocycle and a phenylpyridylcarbazole macrocycle was synthesized in yield up to 75%. Single-crystal structures and theoretic computation uncovered that the skeleton preorganization promoted the formation of cyclization-favorable conformation of noncyclic precursors *via* π⋯π interactions. This result provided a new approach for the efficient syntheses of macrocycles.

## Abstract 2

Reported here is the efficient macrocyclization facilitated by skeleton preorganization. Pyridylcarbazole macrocycles and one phenylpyridylcarbazole macrocycle was synthesized in yield up to 75%.

## Graphical abstract

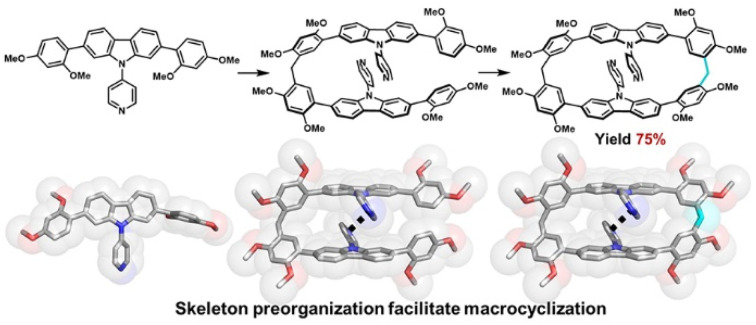

Macrocyclic compounds are important tools for supramolecular chemistry and are widely applied in material chemistry, biomedical chemistry, photochemistry, *etc.*^1–9^ Thus, the syntheses of macrocycles are of great importance. Although numerous pioneering works have considerably advanced in decades, the design and syntheses of macrocycles in an efficient approach remain a challenge.^10–19^ In many cases, the further research and applications of macrocycles were severely limited by the low yields. Macrocyclization is generally a yield-determining step. To improve the cyclization yield, a common method is high dilution. In a high-dilution condition, the noncyclic precursors favor intramolecular reaction producing macrocycles but not intermolecular processes leading to noncyclic oligomers/polymers. But dilution-resulted low concentration usually make the large-scale preparation difficult. An alternative strategy is preorganization, which can be classified as template-assisted preorganization and intramolecular intrinsic preorganization.^20^ For the former, the template should interact with reaction precursor by noncovalent interactions in sufficient strength. A typical example is the synthesis of crown ether in which the alkaline metal cation as template binds the oxygen atoms of the noncyclic precursor and closes the reactive sites for better cyclization.^17^ In addition to alkaline metal cation, the templates can be acids, solvents, aromatic molecules, and aliphatic ammonium for the syntheses of cucurbit[*n*]urils,^21^ pillar[*n*]arenes,^22^ bluebox,^23^ and other macrocyclic arenes.^24^ For the intramolecular intrinsic preorganization, noncyclic precursors/intermediates adopt conformations favoring the intramolecular cyclization and disfavoring the intermolecular polymerization by bringing two ends of open-chain precursors into close proximity *via* noncovalent interactions. The exemplification are the syntheses of peptide macrocycles and cyclic oligoamides promoted by hydrogen bond.^25,26^ The intramolecular intrinsic preorganization *via* other noncovalent interactions is rarely reported. Herein we report an intramolecular skeleton preorganization for efficient macrocyclization by preorganizing the noncyclic precursors *via* π⋯π interactions.

The skeleton extended pyridylcarbazole monomer (PyCM) was synthesized by coupling the carbazole monomer (CM) with 4-bromopyridine *via* a Buchwald–Hartwig reaction in 85% yield (Scheme S1, and Fig. S1–S3†). We first tried a commonly used cyclization condition: BF_3_·Et_2_O as catalyst, 1,2-dichloroethane (DCE) as solvent and at 25 °C.^27^ Intriguingly, a cyclic dimer (PyC[2]) was obtained in 23% yield with trace cyclic trimer (PyC[3]) and tetramer (PyC[4]), which were detected in the crude mixture (Fig. S4–S8†). Since related linear monomers and another slightly bent carbazole monomer were previously reported to form cyclic trimers,^27,28^ the unusual dimeric cyclic product PyC[2] led us to comprehensively screen reaction conditions to determine the origin of this selectivity (Scheme 1, and Table S1†). Catalysts are crucial to the cyclization reaction, therefore Brønsted acids and Lewis acids were firstly evaluated. For Brønsted acids, strong acid TfOH gave 75% yield of PyC[2] (Entry 1), moderate-strong acids showed yields of 37% for *p*-TsOH and 12% for CF_3_COOH (entries 3 and 5), while weak acid (CH_3_COOH) was unable to catalyzing this reaction even large excess was added (Entry 6). The decrease of catalysts ratio sharply reduced yields from 75% to 58% for TfOH (entries 1 and 2), and from 37% to 0% for *p*-TsOH (entries 3 and 4). These results proved that only strong and moderate-strong Brønsted acids work for this cyclization reaction and adequately catalysts are essential. Lewis acids showed low to moderate yields (7% for AlCl_3_, 43% for FeCl_3_, 23% for BF_3_·Et_2_O) (Table S1,† entries 7–9). Therefore, TfOH is the best catalyst among Brønsted acids and Lewis acids.

**Scheme 1:**
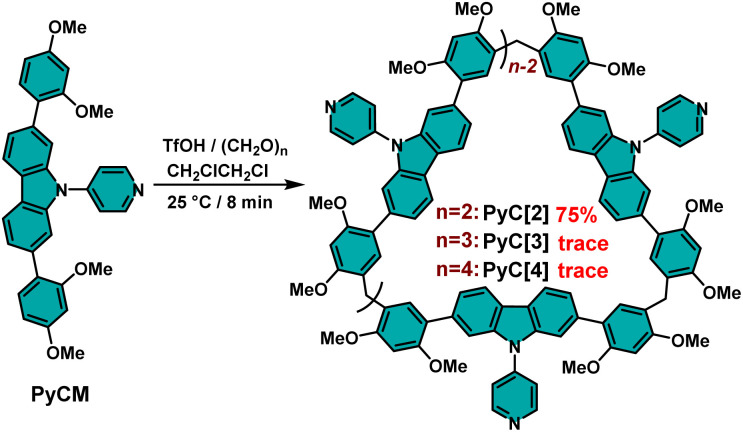
The syntheses of pyridylcarbazole macrocycles (PyC[2–4]).

Furthermore, the influence of solvents was elucidated. In the optimal catalyst ratio, the reaction showed yields of 10% in 1,1,2,2-tetrachloroethane (TCE), 49% for DCM, 72% for CHCl_3_, and 30% for CH_3_CN, lower than that of in DCE (75%), which proved that the best solvent is DCE (entries 10–13). Moreover, the reaction temperature was also important: elevating temperature would lower the yield (62% for 40 °C and 54% for 60 °C, entries 15–16), while the reaction was halted when the temperature was decreased to 0 °C (entry 14), which proved the optimal temperature is 25 °C. The best reaction time was about 8 min and prolonging the reaction time sharply decreased the yield of PyC[2] to 0% and generated byproducts of oligomers and polymers (entries 1, and 17–20). Therefore, the optimum condition was TfOH as catalyst, DCE as solvent and at 25 °C. In most reaction conditions, PyC[2] is the dominant product. Generally, the main product of a cyclization reaction is structural stable and energy favorable. This unusual result indicated that there must be some noncovalent interactions to facilitate the formation of cyclic dimer.

To reveal the origin of the size-selective macrocyclization in atomic-level structures, single-crystal X-ray diffraction analysis were performed. Single crystals of PyC[2] were obtained as colorless needle by slow diffusing *n*-hexane into a DCM solution of PyC[2] during two weeks (Fig. 1a and Table S2†). Unfortunately, better crystal data were unavailable after multiple attempts and the relatively poor crystalline quality made it hard to precisely measure the intra-/intermolecular distances, but there were still valuable information about its connectivity and configuration. Intriguingly, two pyridylcarbazole units stacked in a head-to-tail mode and each pyridyl moiety pointed at the carbazole units with edge-to-face π⋯π interactions. As aforementioned, the cyclization propensity of noncyclic precursors is mainly determined by the appropriate conformation caused favorable preorganization. This head-to-tail stacking mode should be the appropriate conformation of the noncyclic dimer which is the precursor of PyC[2]. We deduced that the π⋯π interactions of skeleton fixed the conformation of noncyclic dimer and boosted the cyclization reaction (Fig. 1b). The π⋯π interactions should be stronger in the reaction conditions because the pyridyl moiety could associate with catalysts to form a more electron-deficient species (*i.e.* protonated compound).

**Fig. 1:**
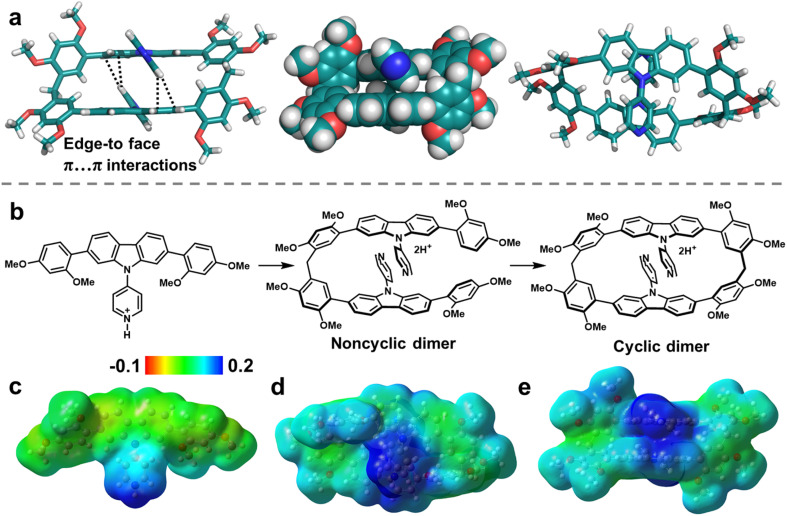
(a) The single-crystal structure of PyC[2]. (b) Cyclization process of PyC[2]. (c–e) Electrostatic potential (ESP) distribution on the (c) protonated PyCM and (d and e) protonated PyC[2]. (Note: cyan; O, red; N, blue; H, white. Red region represents low potential area with the characterization of an abundance of electrons. Blue region represents high potential area with the characterization of a relative absence of electrons).

This was further proved by the electrostatic potential (ESP) distribution on the protonated PyC[2] and PyCM mapped by the density functional theory (DFT) calculations (Fig. 1c–e). Both of them displayed positive ESP in the pyridyl moiety and negative ESP in the carbazole unit. The regions of opposite ESP provided additional interaction for synergistic adjusting the noncyclic dimer into an appropriate conformation with the π⋯π interactions involved skeleton preorganization. Furthermore, we halted the cyclization reaction and got the noncyclic dimer in 37% yield, proved the formation of noncyclic dimeric precursor (Fig. S9–S11†). Therefore, a cyclization mechanism was proposed. Firstly, the monomer was protonated by TsOH and further formed protonated noncyclic dimer by condensing with paraformaldehyde. Secondly, the noncyclic dimer preorganized into a head-to-tail conformation by skeleton preorganization and then fast cyclized to produce the PyC[2]. In this case, the skeleton preorganization played a critical importance in the cyclization.

With this positive result in mind, we further extended the monomer skeleton to verify the mechanism. Phenylpyridylcarbazole monomer (BPyCM) was synthesized in 91% yield by a similar procedure with PyCM (Scheme S2, Fig. S12–S14†). Under the optimal conditions (TfOH as catalyst, DCE as solvent and at 25 °C), a cyclic trimer (BPyC[3]) was obtained in 56% yield after 6 hours (Scheme 2, Fig. S15–S17†). Considering the large steric hindrance, this result hinted that there should be some preorganization during the cyclization. The attempt to obtain single crystal was failed, therefore the optimized structure was calculated by using Mopac2016 program based on the single crystal of carbazole macrocycle.^27^ As illustrated in Scheme 2, typical edge-to-face π⋯π interactions were found with distances of 2.82 and 2.58 Å, which contributed to the skeleton preorganization.

**Scheme 2:**
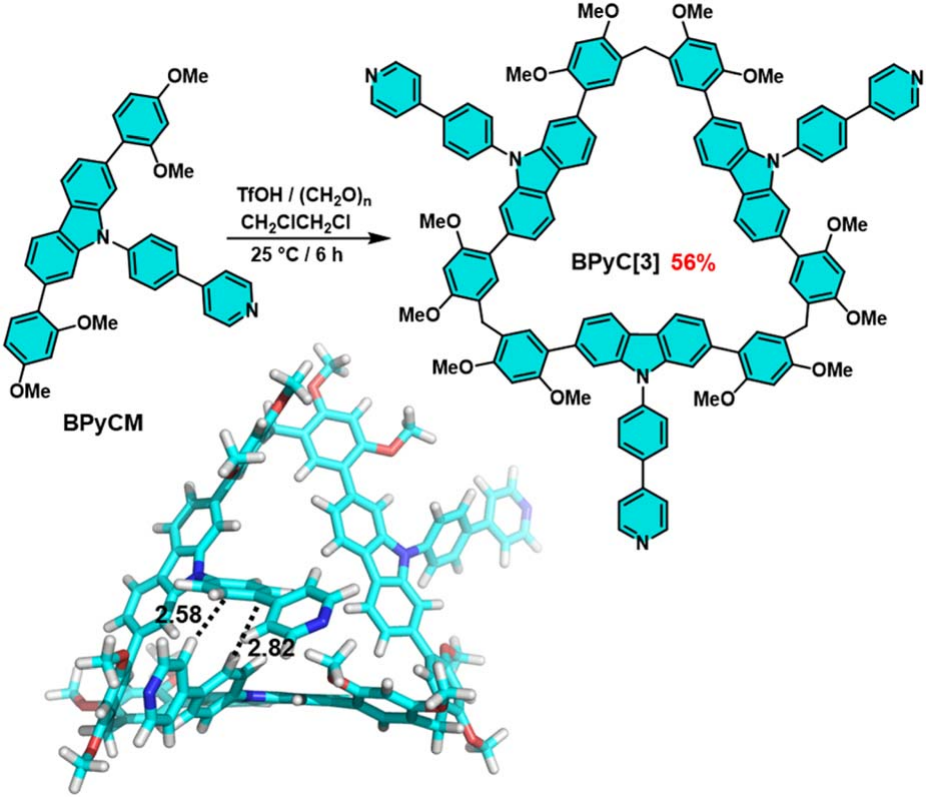
The synthesis and the optimized structure of BPyC[3]. Note: cyan; O, red; N, blue; H, white.

We further explored the photophysical properties of macrocycles and monomers. PyC[2] and PyCM showed same photoluminescence spectra peaks at 400 nm with lifetimes of 0.95 and 1.3 ns, respectively (Fig. 2a, S18a, b, and Table S3†). Their delay time photoluminescence spectra also showed similar peak at 515 nm with microsecond lifetimes (284 μs for PyC[2] and 353 μs for PyCM), revealed phosphorescence emission (Fig. 2a, S18c and d†). The photoluminescence quantum yields are 1.8% for PyC[2] and 5.0% for PyCM (Fig. S19†). The higher photoluminescence quantum yields of monomer probably attributed to the tighter packing, which was confirmed by the single crystal structures. PyCM mutually stacked with multiple edge-to-face and face-to-face π⋯π interactions, confined the molecules and suppressed the nonradiative decay (Fig. 2d, S20, and Table S2†). For PyC[2], limited intermolecular face-to-face π⋯π interactions between 2,4-dimethoxyphenyls and edge-to-face π⋯π interactions between 2,4-dimethoxyphenyls and pyridyls resulted loose stacking and enormous voids, which is unfavorable to the confinement (Fig. 2c, S21, and Table S2†). BPyC[3] showed a photoluminescence spectra peak at 594 nm and a delay time photoluminescence spectra peak at 582 nm with lifetimes of 23 ns and 373 μs, respectively. The monomer BPyCM has a blue-shift photoluminescence spectra (407 nm, *τ* = 1.8 ns) and a slightly red-shift delay time photoluminescence spectra (596 nm, *τ* = 380 μs) with a higher photoluminescence quantum yield comparing with macrocycle (11% for BPyCM, and 7.5% for BPyC[3]) (Fig. 2b, S22, S23, and Table S3†). All results indicated that the photophysical properties of these compounds were probably depended on the intermolecular interactions in the solid state.

**Fig. 2:**
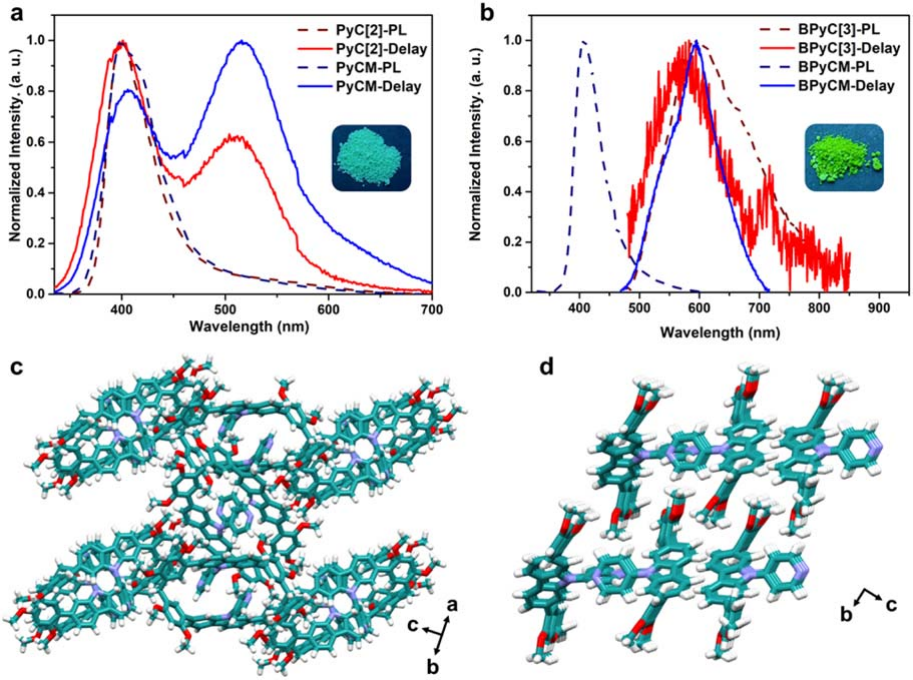
(a) Photoluminescence (dash line) and delay time photoluminescence spectra (solid line) of PyC[2] (red) and PyCM (blue) in the solid state; (b) Photoluminescence (dash line) and delay time photoluminescence spectra (solid line) of BPyC[3] (red) and BPyCM (blue) in the solid state ((Inset) luminescence photographs of macrocycles under 254 nm light); (c) the solid-state superstructure of PyC[2]; (d) the solid-state superstructure of PyCM. Note: cyan; O, red; N, blue; H, white.

In conclusion, we have demonstrated the effective promotion of skeleton preorganization for the macrocyclization. The skeleton preorganization can adjust the non-cyclic precursors into a cyclization-favorable conformation by head-to-tail π⋯π interactions and therefore facilitate the macrocyclization. As a result, a pyridylcarbazole macrocycle PyC[2] was synthesized in yield up to 75% and a phenylpyridylcarbazole macrocycle BPyC[3] was synthesized in 56% yield. Moreover, these macrocycles and monomers showed fluorescence and phosphorescence with photoluminescence quantum yield up to 11%, which depended on the intermolecular interactions in solid state. This finding proved the positive and important role of skeleton preorganization in cyclization reaction and would contribute to the design and syntheses of macrocyclic compounds.

## Author contributions

Zhi-Yuan Zhang, Ming Dong, conceptualization; Rong-Yao Xu, Xiu Liu, Guang Sun, data curation; Liya Zhao, Si-Miao Zhang and Xi-Yang Wang, formal analysis; Rong-Yao Xu, Xiu Liu, Hong-Xu Zhang, Shang-Jie Yang, Xiuguang Wang, investigation; Rong-Yao Xu, writing – original draft; Zhi-Yuan Zhang, writing – review & editing; Chunju Li, supervision; Bin Li, Jian Li, project administration; Zhi-Yuan Zhang, Ming Dong, Bin Li, Rong-Yao Xu, Jian Li, Chunju Li, funding acquisition.

## Conflicts of interest

There are no conflicts to declare.

## Supplementary Material

RA-013-D3RA05671F-s001

RA-013-D3RA05671F-s002
