# Supplementary material for: Efficient macrocyclization facilitated by skeleton preorganization
Source: RSC Adv. 2023 Oct 24;13(43):30269–72. doi: 10.1039/d3ra05671f (PMC10594055; doi:10.1039/d3ra05671f)
Supplement: RA-013-D3RA05671F-s001 [file RA-013-D3RA05671F-s001.pdf]

Electronic Supplementary Material (ESI) for RSC Advances.
This journal is © The Royal Society of Chemistry 2023

Supporting Information

Efficient Macrocyclization Facilitated by Skeleton Preorganization

Rong-Yao Xu+,[a] Xiu Liu+,[a] Guang Sun+,[a] Zhi-Yuan Zhang,,[a] Ming Dong*,[a] Liya Zhao,[a] Si-Miao Zhang,[a] Xi-Yang Wang,[a] Hong-Xu Zhang,[a] Shang-Jie Yang,[a] Xiuguang Wang,[a] Bin Li,[a] Jian Li,[b] and Chunju Li*,[a]*

a. Tianjin Key Laboratory of Structure and Performance for Functional Molecules, College of Chemistry, Tianjin Normal University, Tianjin 300387 (P. R. China).

b. School of Chemistry and Chemical Engineering, Henan Normal University, P. R. China.

Corresponding Author

*E-mail:
zzy@tjnu.edu.cn
hxxydongm@tjnu.edu.cn
cjli@shu.edu.cn

Table of Contents

Section I. Materials/Methods/Instrumentation 2

Section II. Synthetic Protocols of PyC[2-4] and BPyC[3]...........................3-13

Section III. Photophysical Properties and Single-crystal Data 14-18

Section IV. References 18

Section I. Materials/Methods/Instrumentation

All reagents and solvents were commercially available and used without further purification, unless otherwise noted. ^{1}H and ^{13}C NMR spectra were recorded on Bruker Avance III 400 MHz. High-resolution mass spectra (HRMS) were determined on Agilent Technologies 6520 Accurate-Mass Q-TOF LC/MS in methods of Dual ESI ion source in positive ion polarity. Fluorescence spectra and lifetimes were measured on FLS1000. Photoluminescence quantum efficiencies were measured on HAMAMATSU C9920-02 by absolute method.

Suitable crystals were selected and on Bruker D8 VENTURE and Bruker SMART APEX II diffractometer. The crystals were kept at 150.15 K for PyC[2] and 170.0 K for PyCM during data collection. Using Olex2[1], the structure was solved with the SHELXT[2] structure solution program using Intrinsic Phasing and refined with the SHELXL[3] refinement package using Least Squares minimisation. We constrained the thermal vibrations of C25 and C28, and averaged electron cloud density of N2, C33, C61 and C62 during the refinement of PyC[2]. Because of the absence of strong interactions between PyC[2] and solvents, the solvents were dispersed in the crystal without fixed positions, therefore solvents were removed during the refinement for better crystal data.

The geometry optimization of protonated **PyC[2]** and **PyCM** and **BPyC[3]** were performed by using Mopac2016 program[4] with PM7[5] level. The electrostatic potential (ESP) distribution were calculated by the density functional theory (DFT) calculations in B3LYP/6-31+G(d, p) level by Gaussian 09 program.

Section II. Synthetic Protocols of PyC[2-4] and BPyC[3]

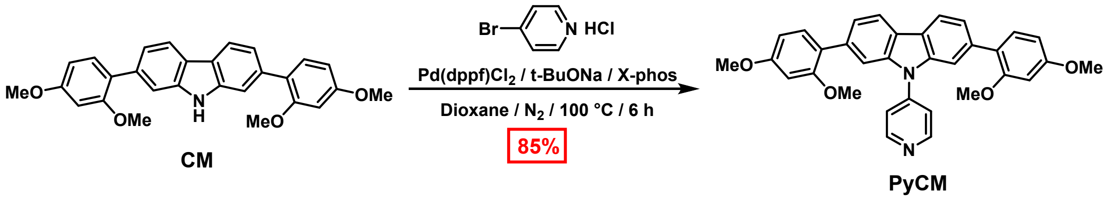

Scheme S1. Synthesis of pyridylcarbazole monomer (**PyCM**).

General procedure: Carbazole monomer **CM** (0.50 g, 1.1 mmol),[6] 4-bromopyridine hydrochloride (0.44 g, 2.3 mmol), Pd(dppf)Cl_2 (42 mg, 0.055 mmol), X-phos (0.11 g, 0.22 mmol), and *t*-BuONa (0.55 g, 5.5 mmol) were added to a dry 1,4-dioxane (50 mL), which had been degassed with N_2 for 15 min. The solution heated to 100 ºC under N_2 for 6 h. Then, the reaction mixture was cooled to room temperature and the organic solvent was evaporated under vacuum. The resulting mixture was extracted with CH_2Cl_2 (3 × 50 mL) and then washed with water and brine (60 mL). The organic layer was dried over anhydrous Na_2SO_4 and evaporated. The residue was purified by column chromatography with CH_2Cl_2: EA (4:1, V:V) as eluent to afford **PyCM** as white powder (0.48 g, 85%).

PyCM: ^{1}H NMR (400 MHz, $CDCl_3$): δ (ppm): 8.80 (d, *J* = 6.2 Hz, 2H), 8.11 (d, *J* = 8.1 Hz, 2H), 7.69 (s, 2H), 7.64 (d, *J* = 6.1 Hz, 2H), 7.48 (dd, *J* = 8.1, 1.3 Hz, 2H), 7.31 (d, *J* = 9.0 Hz, 2H), 6.76 – 6.40 (m, 4H), 3.87 (s, 6H), 3.82 (s, 6H). ^{13}C NMR (100 MHz, $CDCl_3$): δ (ppm): 161.33, 158.43, 152.43, 146.72 ,140.77,137.53, 132.51, 124.81, 124.02, 123.77, 121.60, 120.78, 111.56, 105.65, 100.04, 56.60, 56.40. HRMS (m/z): calcd. for $C_{33}H_{30}N_2O_4^+$, 517.2122 [M+H]$^+$; found, 517.2114.

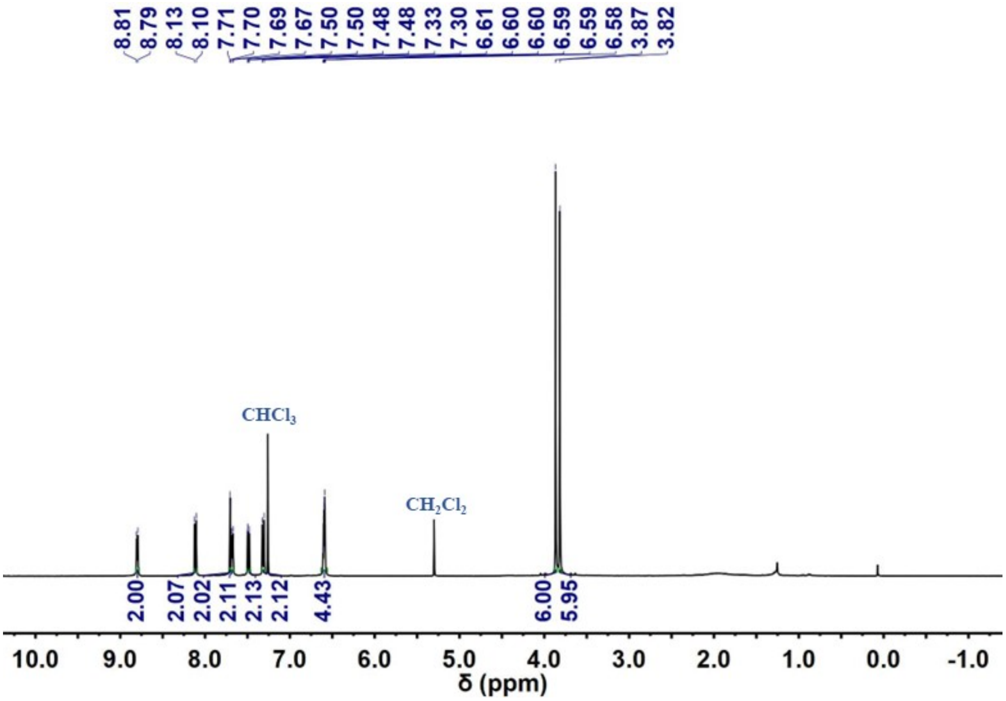

Figure S1. ^{1}H NMR spectrum (400 MHz, $CDCl_3$, 298 K) of **PyCM**.

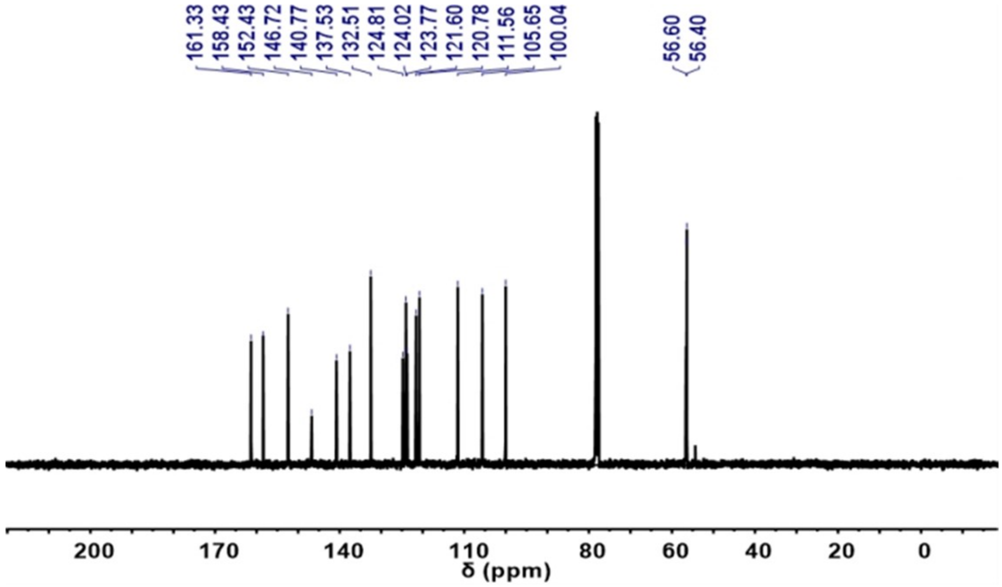

Figure S2. ^{13}C NMR spectrum (100 MHz, $CDCl_3$, 298 K) of **PyCM**.

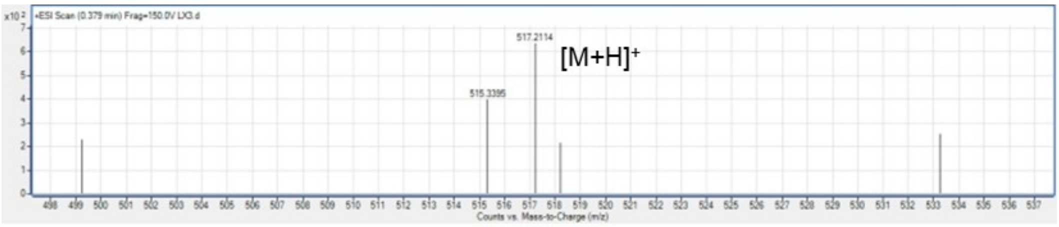

Figure S3. HRMS of **PyCM**.

Syntheses of PyC[2-4].

To the solution of **PyCM** (0.47 g, 0.91 mmol) and paraformaldehyde (0.041 g, 1.37 mmol) in DCE (200 mL) was added TfOH (97 μL, 1.1 mmol) and stirred at 25 °C for 8 minutes. After quenching by 100 mL water, the organic phase was separated and washed with saturated $NaHCO_3$ solution. The organic layer was dried over anhydrous Na_2SO_4 and evaporated. The residue was purified by column chromatography with CH_2Cl_2: EA: MeOH (2:1:0.1, V:V:V) as eluent to afford **PyC[2]** as white powder (0.36 g, 75%) and **PyC[3-4]** can be detected in HRMS.

***PyC[2]*:** ^{1}H NMR (400 MHz, TCE-d_2): δ (ppm): 8.63 (d, J = 6.1 Hz, 4H), 8.01 (d, J = 8.1 Hz, 4H), 7.71 (s, 4H), 7.64 – 7.49 (m, 4H), 7.44 – 7.27 (m, 4H), 7.07 (s, 4H), 6.58 (s, 4H), 3.95 (s, 4H), 3.91 (s, 12H), 3.83 (s, 12H). ^{13}C NMR (100 MHz, TCE-d_2): δ (ppm): 157.60, 155.54, 151.18, 145.30, 139.34, 136.52, 132.12, 122.88, 122.22, 122.10, 121.15, 120.21, 119.63, 110.66, 95.96, 56.01, 55.79, 27.46. HRMS (m/z): calcd. for $C_{68}H_{56}N_4O_8^+$ [M]$^+$, 1056.4098; found, 1056.4095.

***PyC[3]*:** HRMS (m/z): calcd. for $C_{102}H_{84}N_6O_{12}^+$ [M]$^+$, 1585.6184; found,1585.6177.

***PyC[4]*:** HRMS (m/z): calcd. for $C_{136}H_{112}N_8O_{16}^+$ [M]$^+$, 2113.8230; found, 2113.8234.

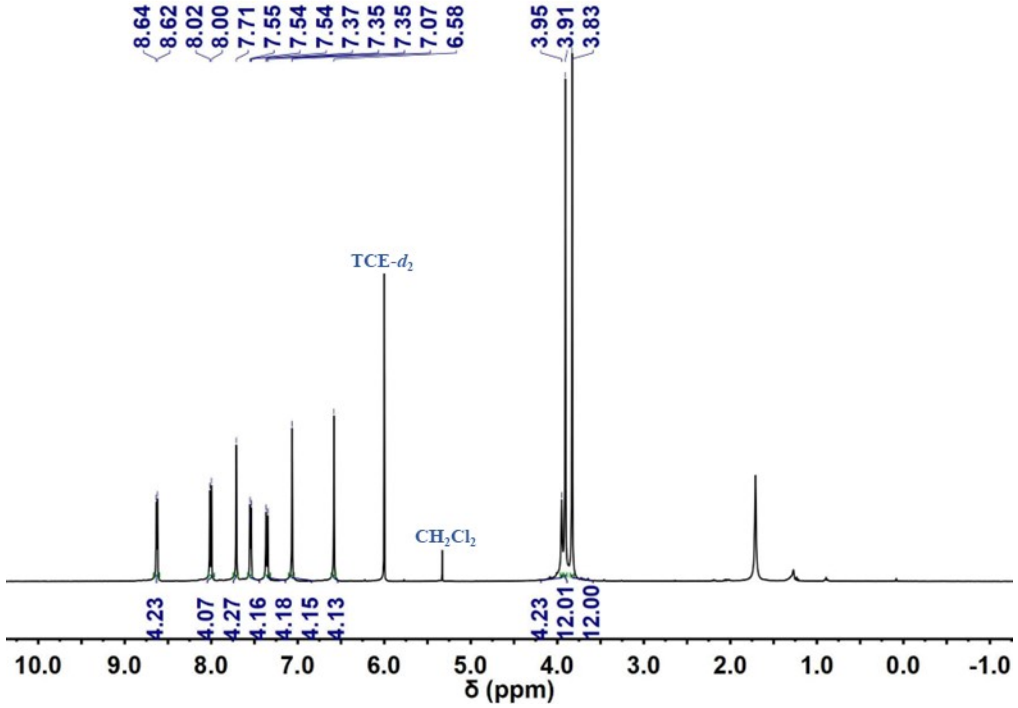

Figure S4. ^{1}H NMR spectrum (400 MHz, TCE-d_2, 298 K) of **PyC[2]**.

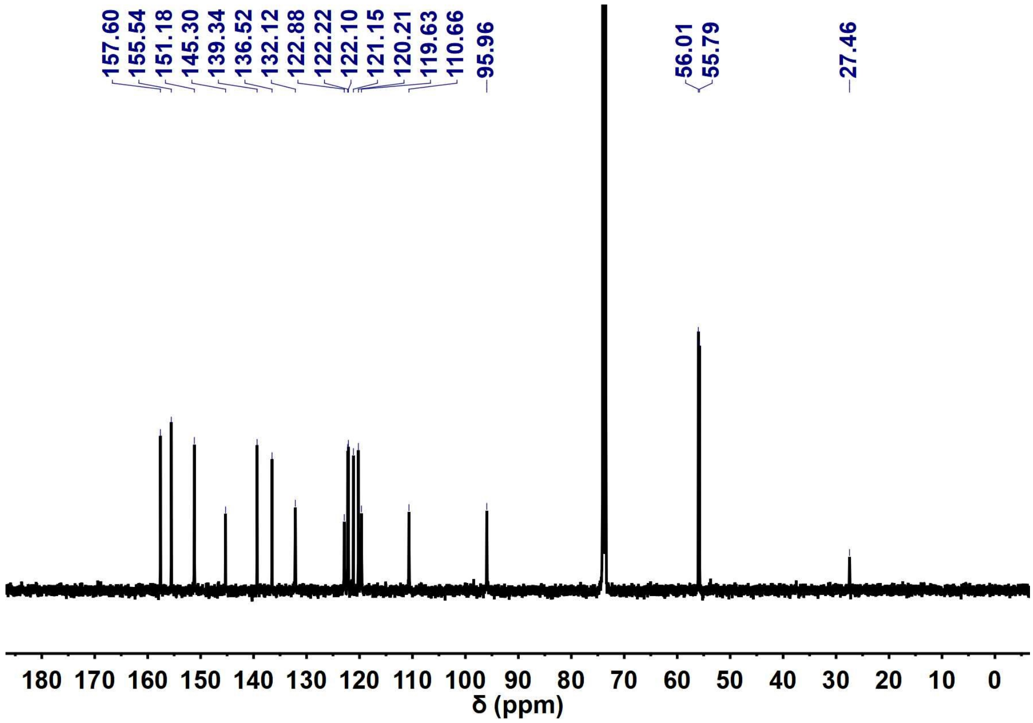

Figure S5. ^{13}C NMR spectrum (100 MHz, TCE-d_2, 298 K) of **PyC[2]**.

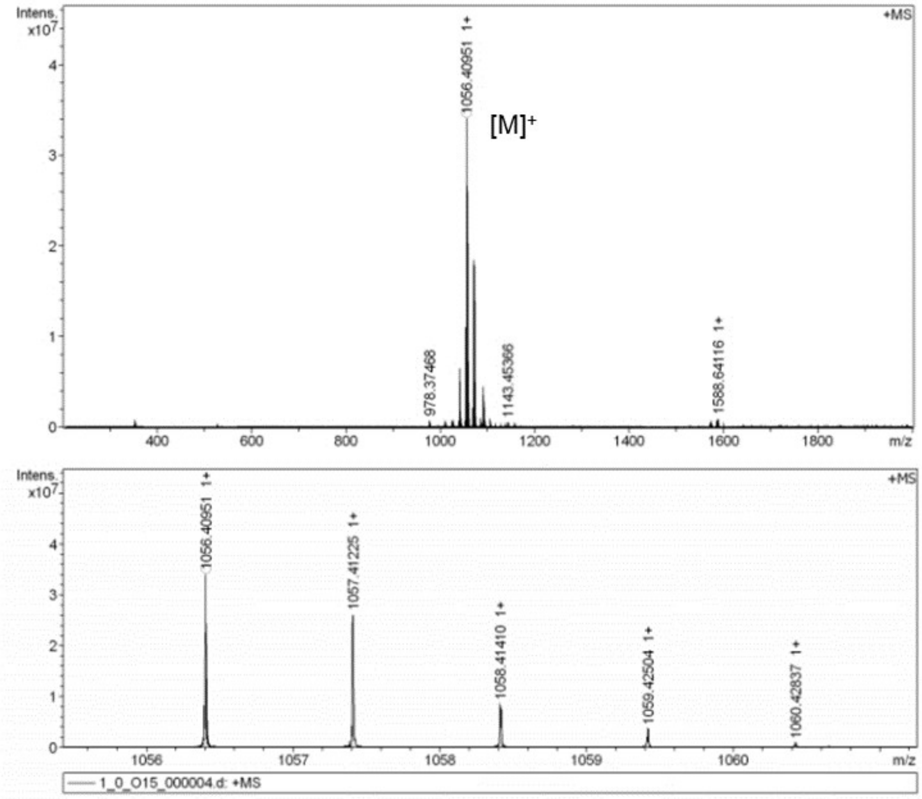

Figure S6. HRMS of **PyC[2]**.

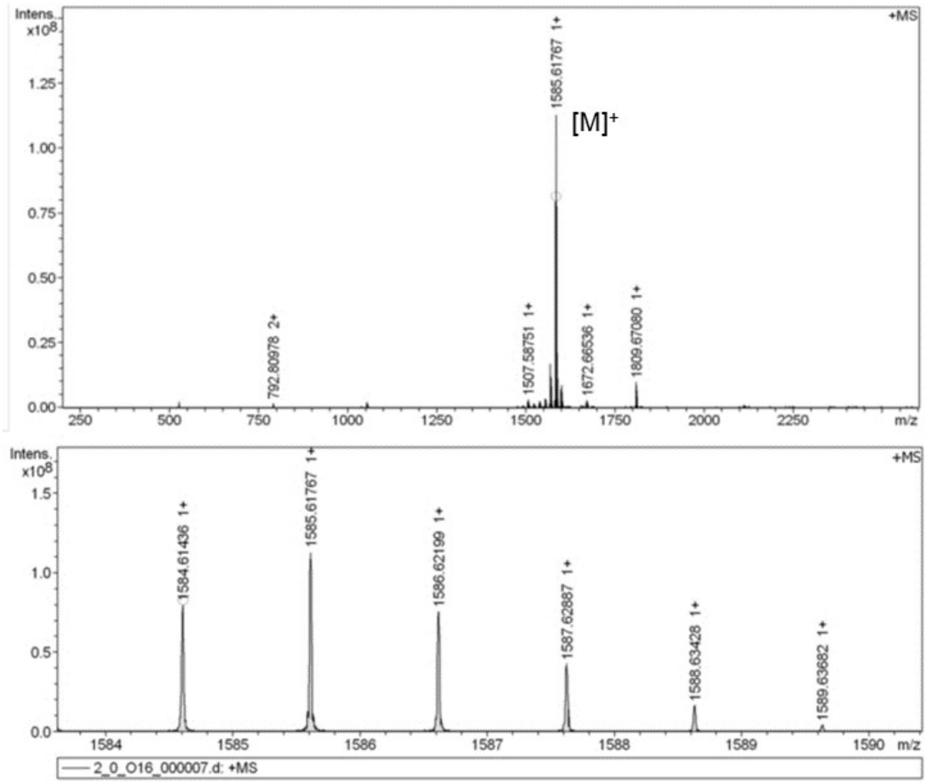

Figure S7. HRMS of **PyC[3]**.

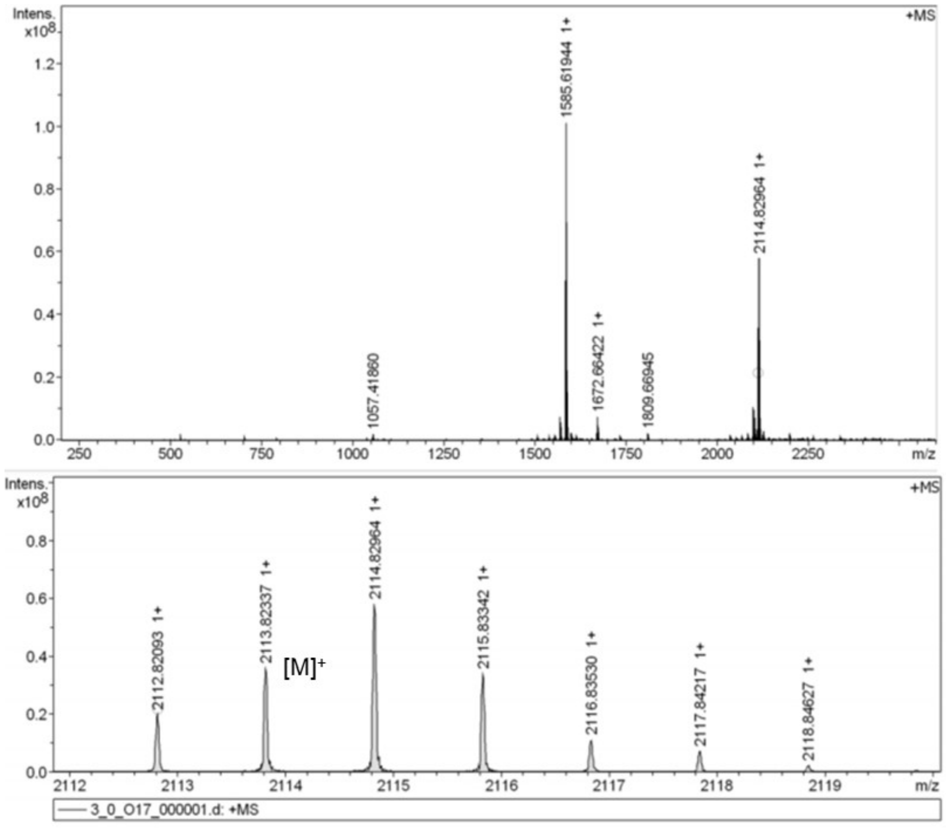

Figure S8. HRMS of **PyC[4]**.

Table S1. Reaction conditions screening for **PyC[2]**.

Entry	Catalysts	Equivalent of Catalysts	Solvents[b]	Temperature (°C)	Reaction time[c]	Yields (%)[d]
1	TfOH	1.2[a]	DCE	25	8 min	75
2	TfOH	0.6	DCE	25	7 h	58
3	*p*-TsOH	30 [a]	DCE	25	30 min	37
4	*p*-TsOH	3.0	DCE	25	10 h	-[e]
5	CF_3COOH	0.67[a]	DCE	25	12 h	12
6	CH_3COOH	315	DCE	25	>12 h	-[e]
7	$AlCl_3$	23[a]	DCE	25	60 min	7
8	$FeCl_3$	4.0[a]	DCE	25	20 min	43
9	$BF_3 \cdot Et_2O$	4.2[a]	DCE	25	20 min	23
10	TfOH	4.7[a]	TCE	25	30 min	10
11	TfOH	5.8[a]	DCM	25	40 min	49
12	TfOH	1.2 [a]	$CHCl_3$	25	20 min	72
13	TfOH	7.0[a]	CH_3CN	25	30 min	30
14	TfOH	1.2	DCE	0	20 min	-[e]
15	TfOH	1.2	DCE	40	2 min	62
16	TfOH	1.2	DCE	60	1.5 min	54
17	TfOH	1.2	DCE	25	40 min	61
18	TfOH	1.2	DCE	25	60 min	50
19	TfOH	1.2	DCE	25	6 h	43
20	TfOH	1.2	DCE	25	24 h	-[e]

[a] The ratio of catalyst is optimal for given conditions. We change the ratios of catalysts and monitor the reaction to determine a best ratio; [b] DCE: 1,2-dichloroethane; DCM: dichloromethane; TCE: 1,1,2,2-tetrachloroethane; CH_3CN; $CHCl_3$; [c] The reaction time is optimal for given conditions; [d] Yield of isolated product; [e] Not detected.

Syntheses of noncyclic dimer

To the solution of **PyCM** (0.47 g, 0.91 mmol) and paraformaldehyde (0.041 g, 1.37 mmol) in DCE (200 mL) was added TfOH (97 μL, 1.1 mmol) and stirred at 25 °C for 5 minutes. After quenching by 100 mL water, the organic phase was separated and washed with saturated $NaHCO_3$ solution. The organic layer was dried over anhydrous Na_2SO_4 and evaporated. The residue was purified by column chromatography with CH_2Cl_2: EA: MeOH (2:1:0.1, V:V:V) as eluent to afford noncyclic dimer as white

powder (0.40 g, 85%).

***Noncyclic dimer*:** ^{1}H NMR (400 MHz, TCE-d_2): δ 8.82 (s, 4H), 8.11 (dd, J = 8.1, 5.5 Hz, 4H), 7.71 (d, J = 36.6 Hz, 8H), 7.51 (m, 4H), 7.35 (d, J = 8.9 Hz, 2H), 7.19 (s, 2H), 6.64 – 6.58 (m, 4H), 6.56 (s, 2H), 3.92 (s, 2H), 3.89 – 3.77 (m, 24H). ^{13}C NMR (100 MHz, TCE-d_2): δ (ppm): 160.10, 157.59, 157.31, 155.58, 150.91, 139.43, 139.38, 136.91, 136.50, 132.76, 131.50, 123.38, 123.31, 123.23, 122.37, 122.22, 122.20, 121.41, 120.93, 120.57, 119.63, 110.58, 104.69, 99.04, 95.65, 56.03, 55.65, 55.42. HRMS (m/z): calcd. for $C_{67}H_{58}N_4O_8^{2+}$, 523.2122 [M+2H]$^{2+}$, found, 523.2142; calcd. for $C_{67}H_{56}NaO_8^{+}$, 1067.3990 [M+Na]$^+$, found, 1067.3996.

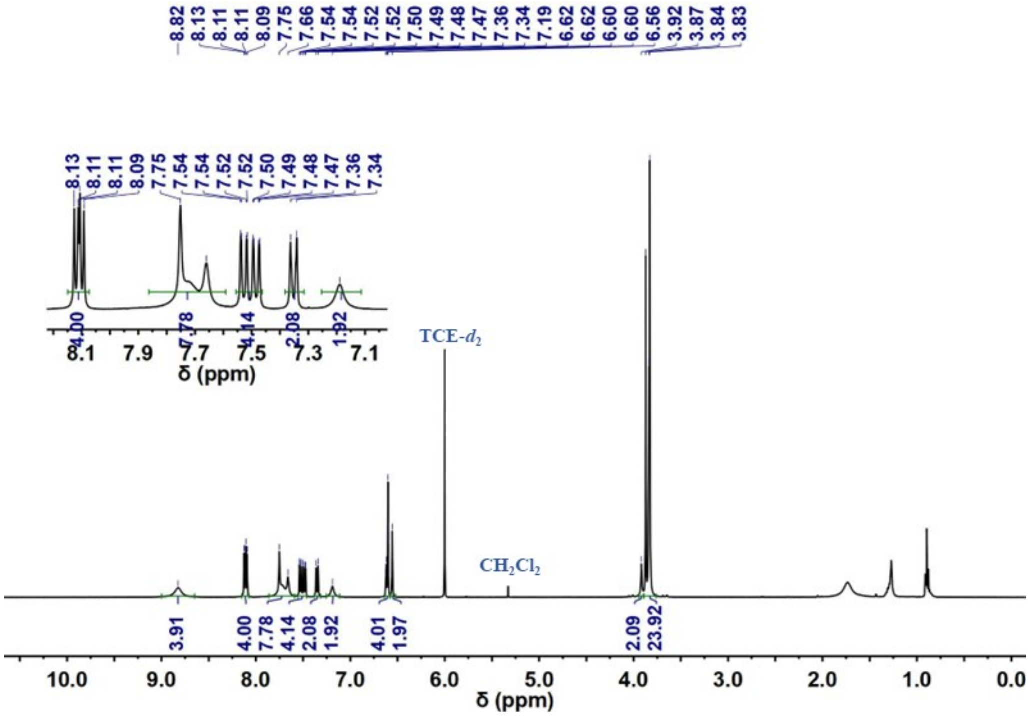

Figure S9. ^{1}H NMR spectrum (400 MHz, TCE-d_2, 298 K) of noncyclic dimer.

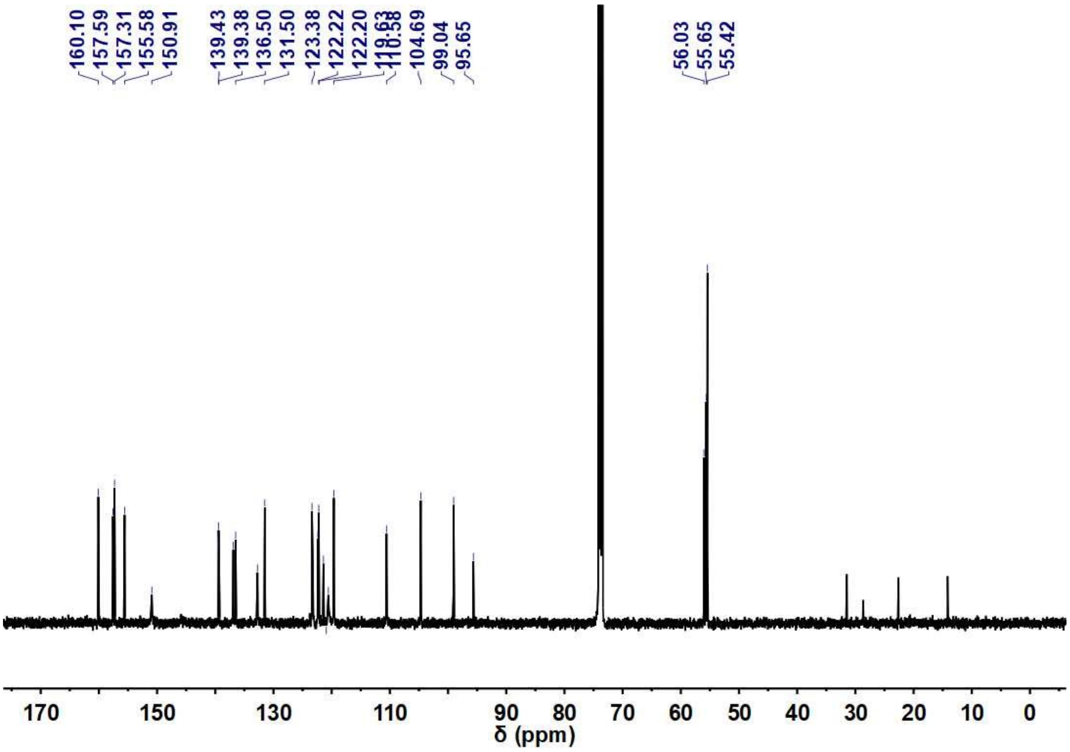

Figure S10. ^{13}C NMR spectrum (100 MHz, TCE-d_2, 298 K) of noncyclic dimer.

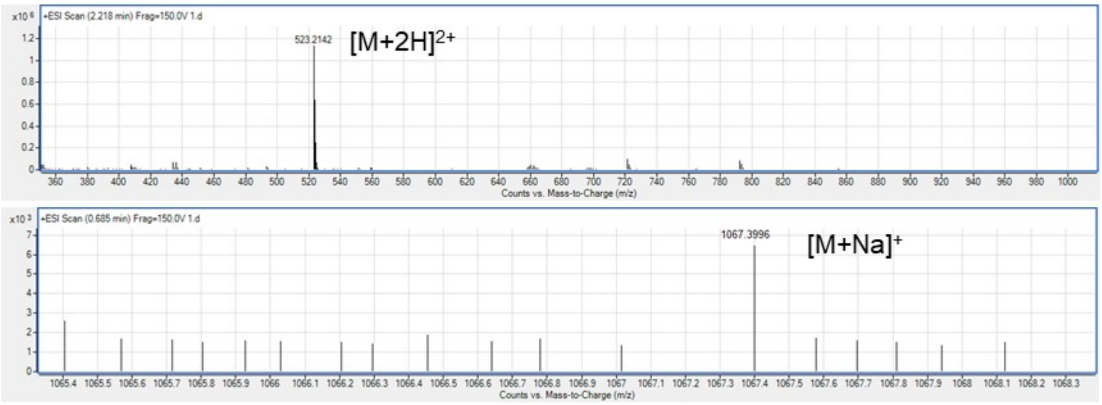

Figure S11. HRMS of noncyclic dimer.

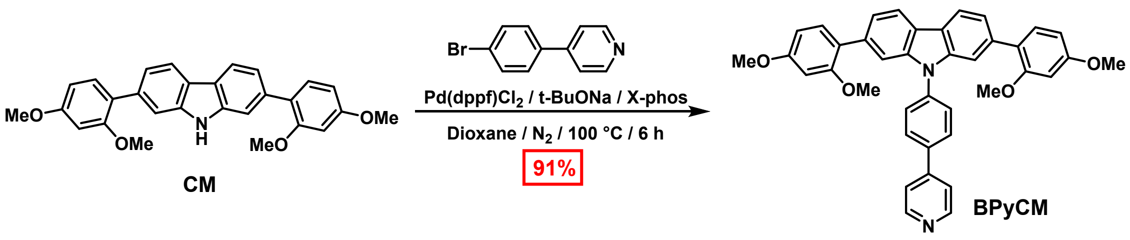

Scheme S2. Synthesis of phenylpyridylcarbazole monomer (**BPyCM**).

The **BPyCM** was synthesized by similar procedure of **PyCM** in yield of 91%.

***BPyCM*:** ^{1}H NMR (400 MHz, $CDCl_3$): δ (ppm): 8.72 – 8.70 (m, 2H), 8.14 (d, *J* = 8.1 Hz, 2H), 7.84 (d, *J* = 8.6 Hz, 2H), 7.76 (d, *J* = 8.6 Hz, 2H), 7.60 – 7.56 (m, 4H), 7.47 (dd, *J* = 8.1, 1.4 Hz, 2H), 7.31 (d, *J* = 8.8 Hz, 2H), 6.59 – 6.55 (m, 4H), 3.85 (s, 6H), 3.81 (s, 6H). ^{13}C NMR (100 MHz, $CDCl_3$): δ(ppm): 160.35, 157.60, 150.41, 147.56, 141.09, 138.96, 136.62, 136.40, 131.68, 128.45, 127.61, 124.32, 122.41, 122.31, 121.63, 119.74, 110.57, 104.73, 99.18, 55.74, 55.52. HRMS (m/z): calcd. for $C_{39}H_{33}N_2O_4^+$, 593.2435 [M+H]$^+$, found, 593.2454.

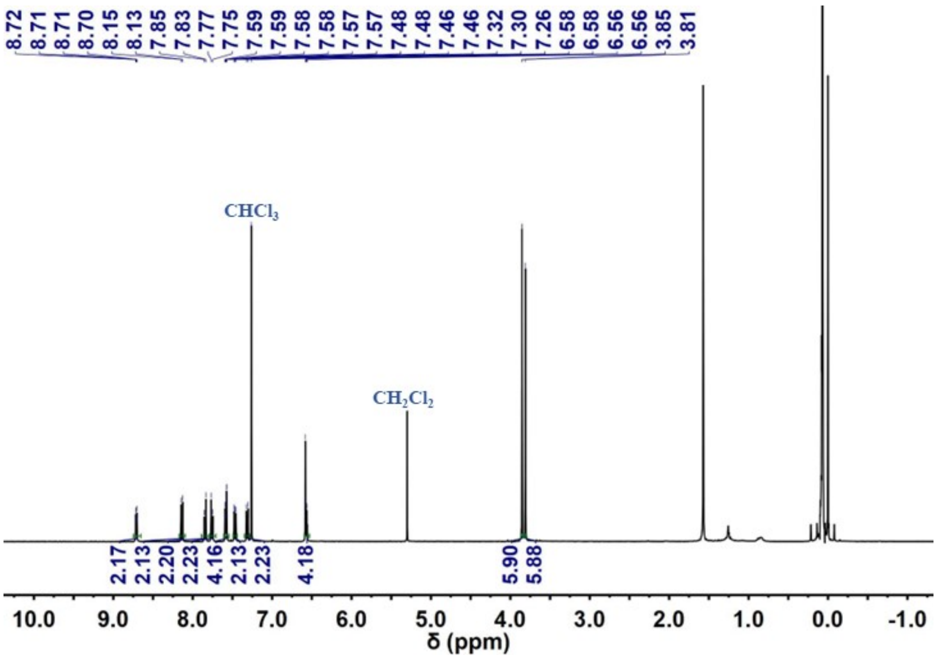

Figure S12. ^{1}H NMR spectrum (400 MHz, $CDCl_3$, 298 K) of **BPyCM**.

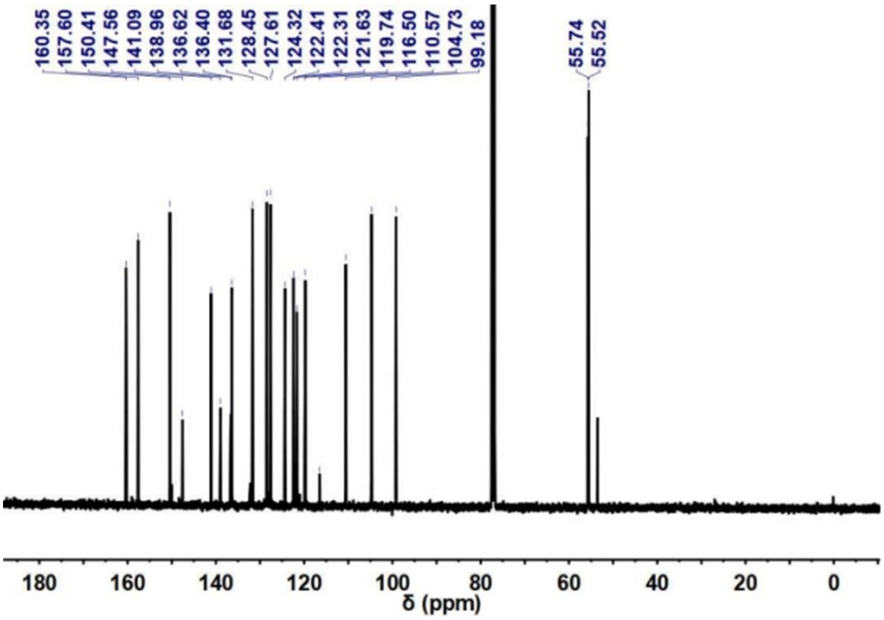

Figure S13. ^{13}C NMR spectrum (100 MHz, $CDCl_3$, 298 K) of **BPyCM**.

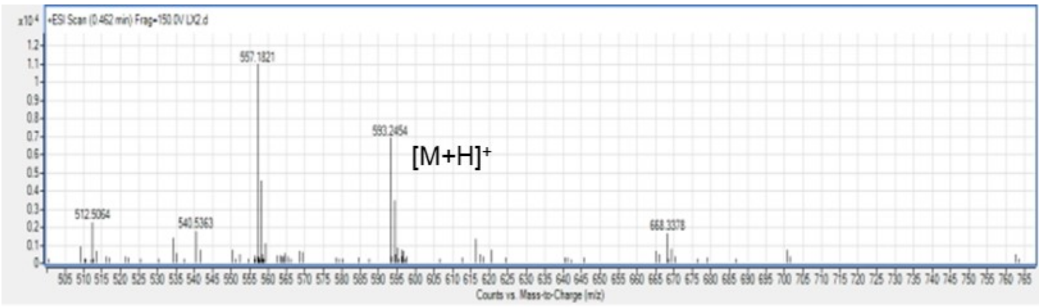

Figure S14. HRMS of **BPyCM**.

Synthesis of BPyC[3].

To the solution of **BPyCM** (0.47 g, 0.79 mmol) and paraformaldehyde (36 mg, 1.2 mmol) in DCE (200 mL) was added TfOH (61 µmL, 0.69 mmol) and stirred at 25 °C for 6 h. After quenching by 100 mL water, the organic phase was separated and washed with saturated $NaHCO_3$ solution. The organic layer was dried over anhydrous Na_2SO_4 and evaporated. The residue was purified by column chromatography with CH_2Cl_2: EA: MeOH (1:1:0.1, V:V:V) as eluent to afford **BPyC[3]** as white powder (0.26 g, 56%).

***BPyC[3]*:** ^{1}H NMR (400 MHz, $CDCl_3$): δ (ppm): 8.36 (d, J = 6.1 Hz, 6H), 7.94 (d, J = 8.1 Hz, 6H), 7.43-7.39 (m, 12H), 7.36 – 7.32 (m, 12H), 7.02 (d, J = 6.1 Hz 6H), 6.97 (s, 6H), 6.56 (s, 6H), 3.93 (s, 6H), 3.87 (s, 18H), 3.76 (s, 18H). ^{13}C NMR (100 MHz, $CDCl_3$): δ (ppm): 157.76, 155.65, 149.31, 147.21, 140.31, 138.58, 136.46, 135.67, 132.48, 128.31, 126.44, 123.25, 122.39, 122.03, 121.71, 121.20, 119.51, 110.49, 96.03,

56.04, 55.87, 29.73. HRMS (m/z): calcd. for $C_{120}H_{96}N_6NaO_{12}^+$ [M+Na]$^+$, 1836.7012; found, 1836.7019.

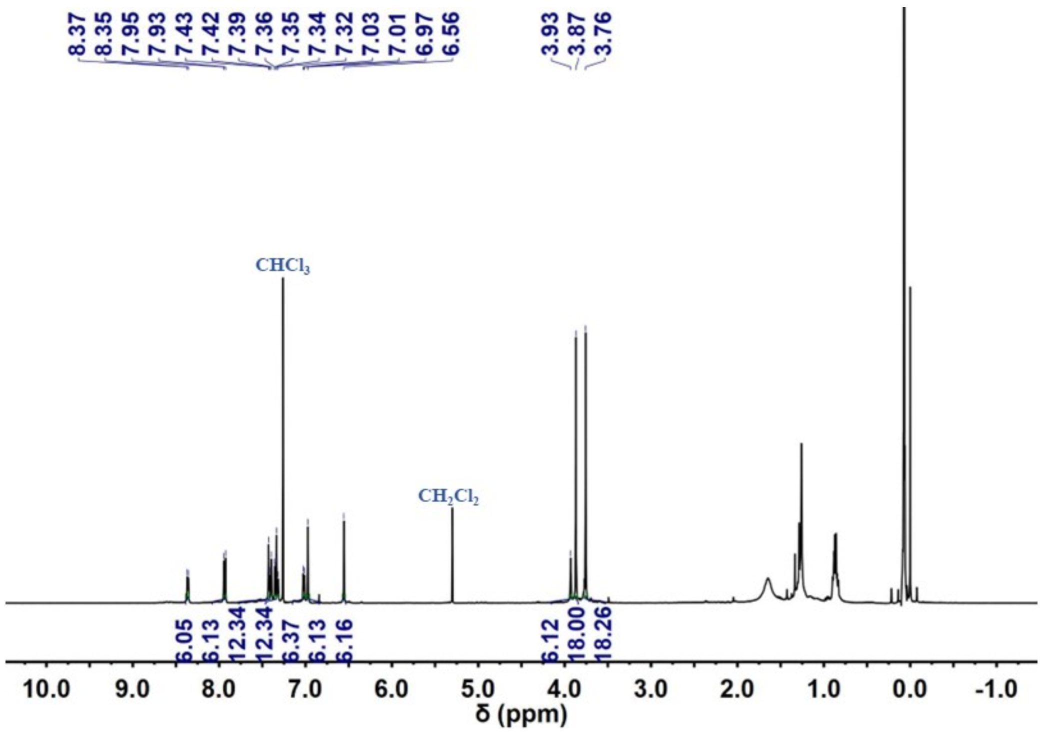

Figure S15. ^{1}H NMR spectrum (400 MHz, $CDCl_3$, 298 K) of **BPyC[3]**.

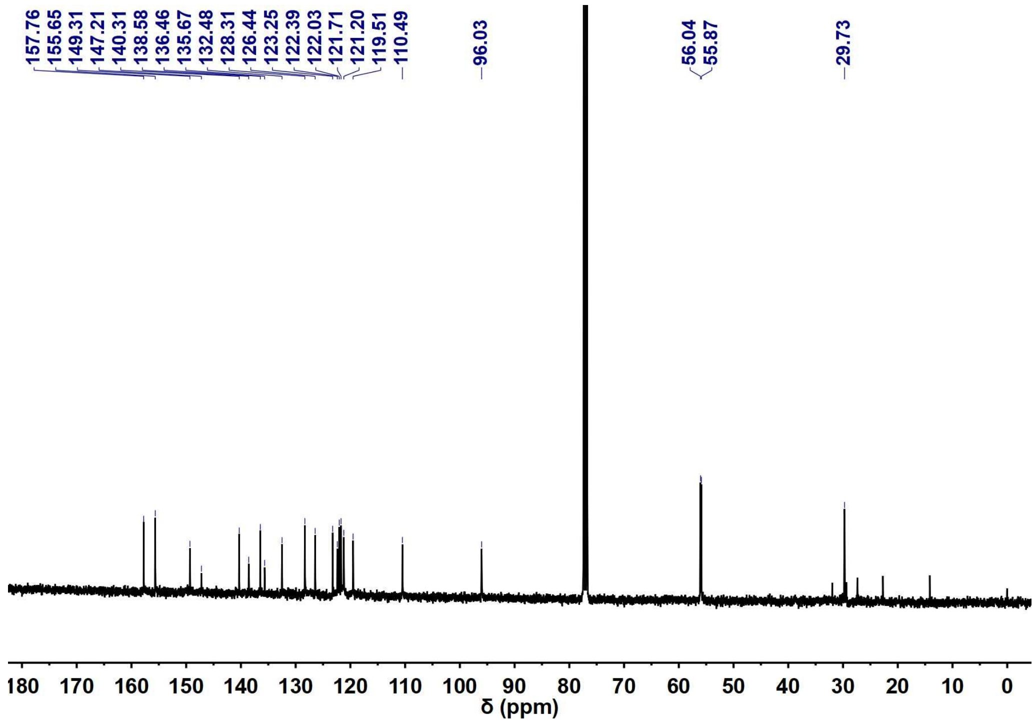

Figure S16. ^{13}C NMR spectrum (100 MHz, $CDCl_3$, 298 K) of **BPyC[3]**.

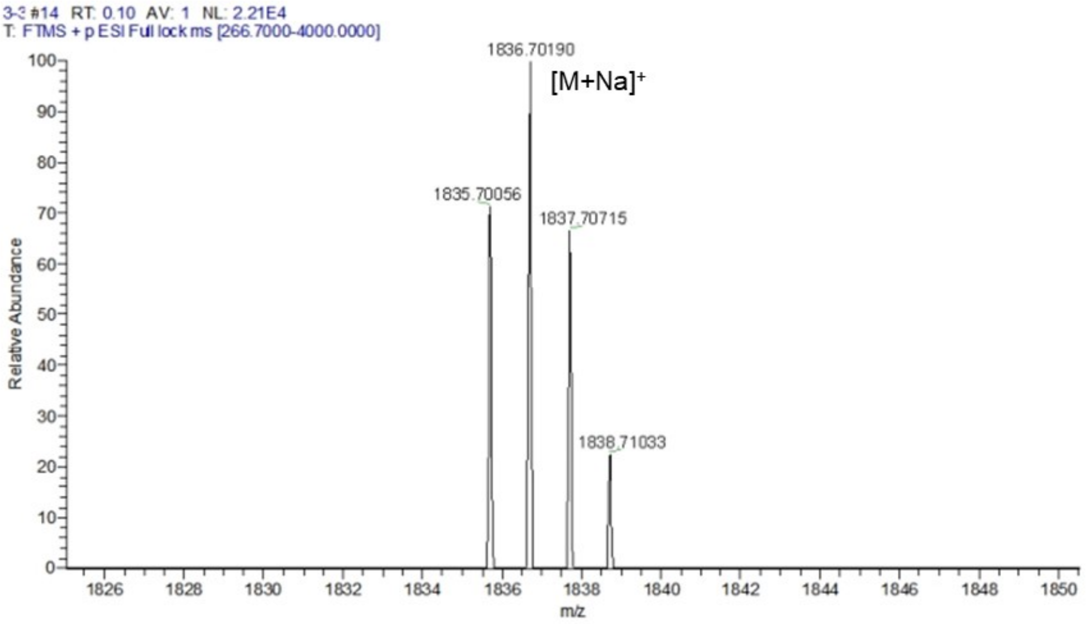

Figure S17. HRMS of **BPyC[3]**.

Section III. Photophysical Properties and Single-crystal Data

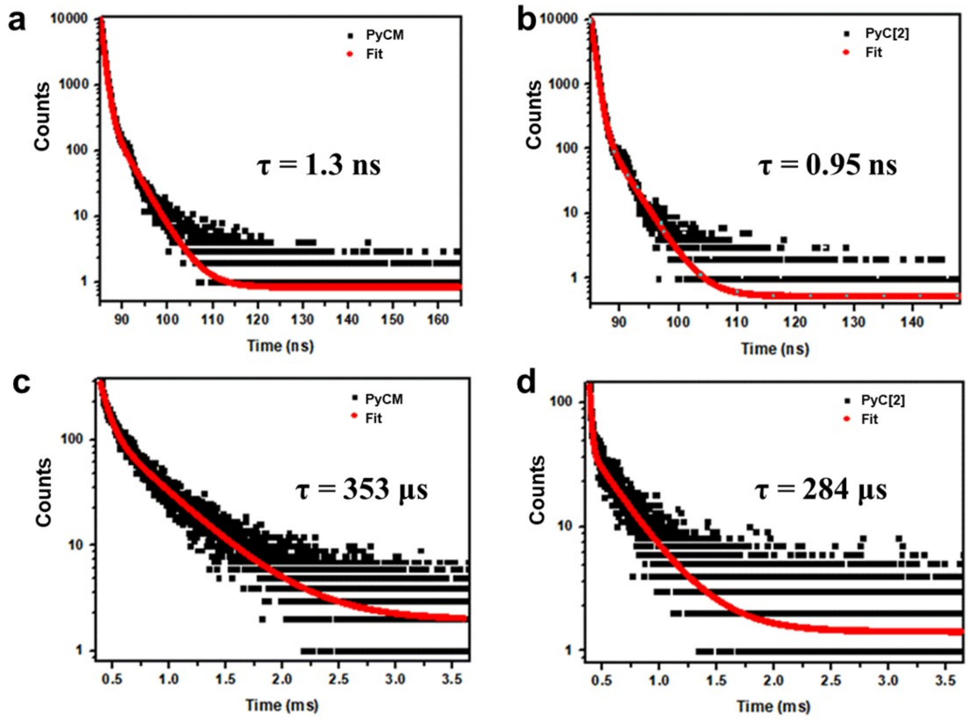

Figure S18. Time-resolved PL decay of (a) **PyCM** (@ 400 nm), (b) **PyC[2]** (@ 400 nm), (c) **PyCM** (@ 515 nm), and (d) **PyC[2]** (@ 515 nm) in the solid state at room temperature.

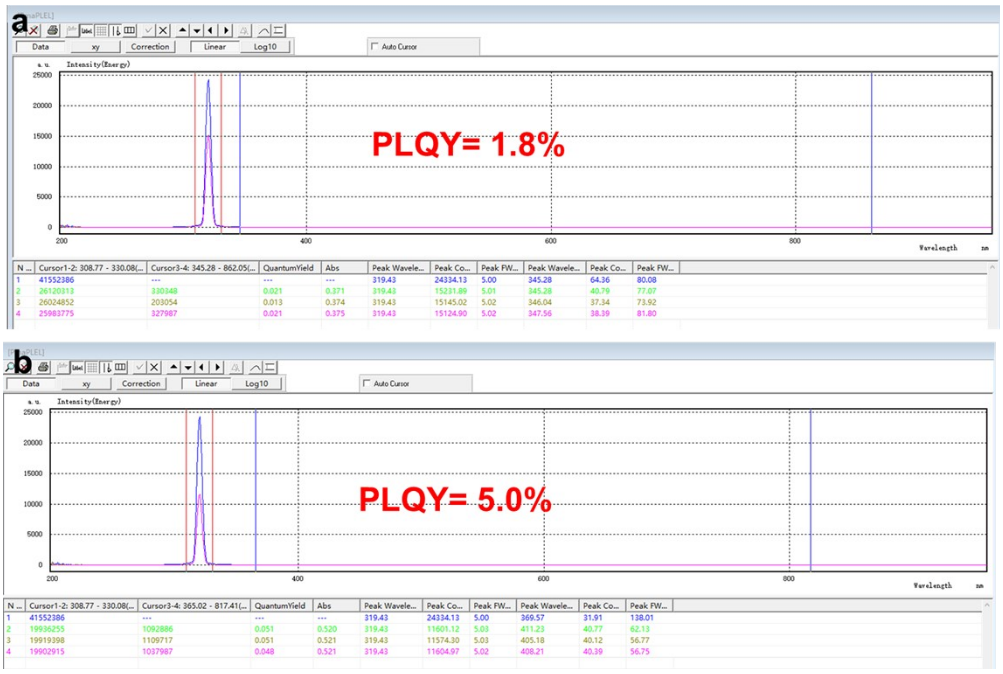

Figure S19. The photoluminescence quantum yields of (a) **PyC[2]** and (b) **PyCM**.

Crystal structure of PyC[2] (CCDC: 2281404) and PyCM (CCDC: 2281407).

Table S2. Crystal data of **PyC[2]** and **PyCM**.

**CCDC number**	**2281404**	**2281407**
Name	**PyC[2]**	**PyCM**
Empirical formula	$C_{68}H_{56}N_4O_8$	$C_{33}H_{28}N_2O_4$
Formula weight	1057.16	516.57
Temperature / K	150.15	170.0
Wavelength/ Å	1.54178	0.71073
Crystal system	monoclinic	triclinic
Space group	C2/c	P -1
a / Å	31.9126(9)	9.126(3)
b / Å	18.7706(7)	11.832(3)
c / Å	24.7876(8)	25.192(6)
α / °	90	98.152(13)
β / °	94.958(2)	95.551(9)
γ / °	90	101.380(10)
Volume/ $Å^3$	14792.7(8)	2617.9(12)
Z	8	4
Density (calculated)	0.949 g/cm^3	1.311 g/cm^3
Absorption coefficient	0.502 mm^{-1}	0.087 mm^{-1}
F(000)	4448.0	1088.0
Crystal size	0.07 × 0.05 × 0.04 mm^3	0.08 × 0.05 × 0.03 mm^3
2θ range for data collection /°	5.468 to 118.276	4.162 to 50.748
Index ranges	$-34 \leq h \leq 35$	$-10 \leq h \leq 10$,
	$-20 \leq k \leq 20$	$-14 \leq k \leq 14$,
	$-27 \leq l \leq 27$	$-30 \leq l \leq 30$
Reflections collected	46774	25859
Independent reflections	10546 [R(int) = 0.1370]	9425 [R(int) = 0.1161]
Data / restraints / parameters	10546/14/730	9425/0/711
Goodness-of-fit on F^2	1.291	1.027
Final R indices [I>2sigma(I)]	R_1 = 0.1283	R_1 = 0.0787
	wR_2 = 0.3731	wR_2 = 0.1437
R indices (all data)	R_1 = 0.1722	R_1 = 0.2065
	wR_2 = 0.4018	wR_2 = 0.2027
Largest diff. peak and hole / e.$Å^{-3}$	0.53/-0.36	0.63/-0.31

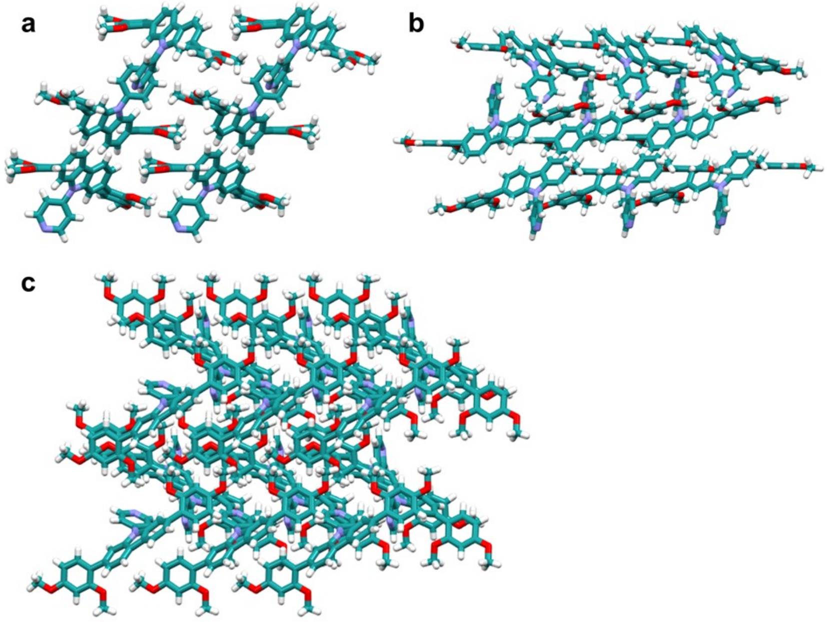

Figure S20. Single-crystal structures and superstructures of **PyCM** view along (a) a axis, (b) b axis, and (c) c axis.

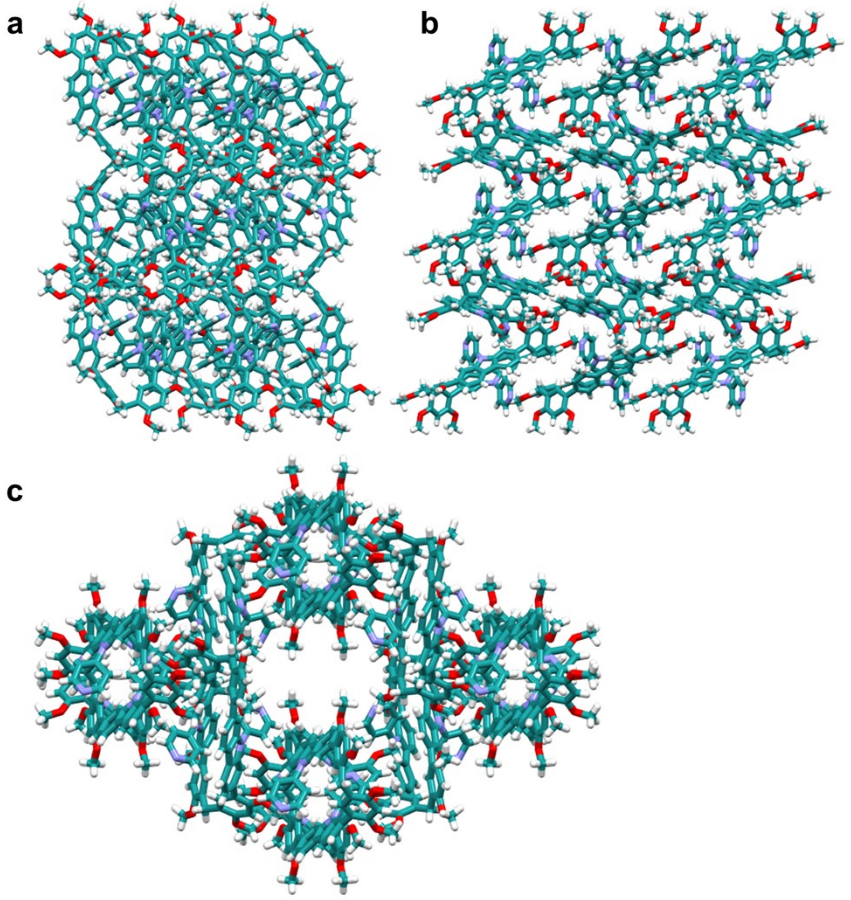

Figure S21. Single-crystal structures and superstructures of **PyC[2]** view along (a) a axis, (b) b axis, and (c) c axis.

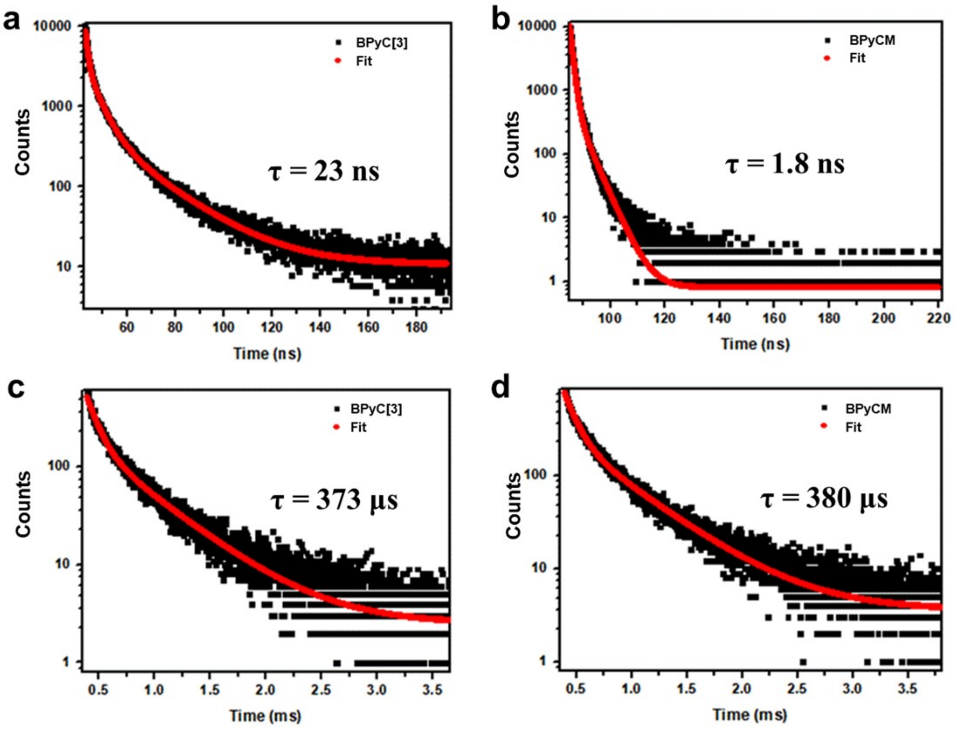

Figure S22. Time-resolved PL decay of (a) **BPyC[3]** (@ 594 nm), (b) **BPyCM** (@ 407 nm), (c) **BPyC[3]** (@ 582 nm), and (d) **BPyCM** (@ 596 nm) in the solid state at room temperature.

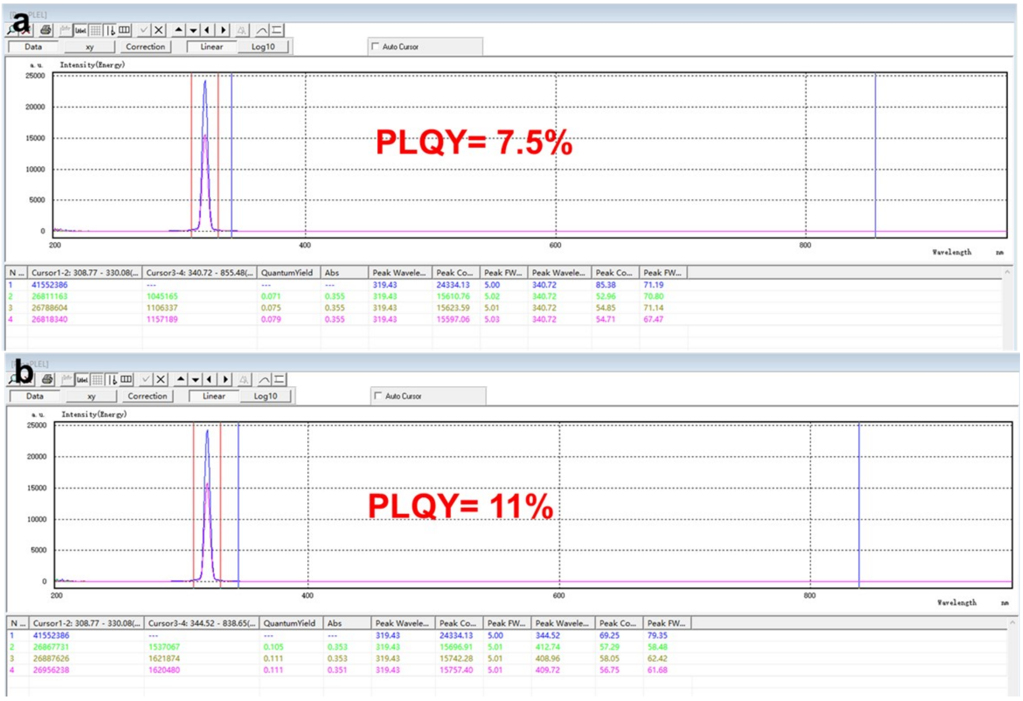

Figure S23. The photoluminescence quantum yields of (a) **BPyC[3]** and (b) **BPyCM**.

Table S3. Photophysical data of skeleton preorganization macrocycles and monomers.

Entry	Compound	λ_F (nm)	λ_P (nm)	τ_F (ns)	τ_P (μs)	Φ_{PL} (%)
1	**PyC[2]**	400	515	0.95	284	1.8
2	**PyCM**	400	515	1.3	353	5.0
3	**BPyC[3]**	594	582	23	373	7.5
4	**BPyCM**	407	596	1.8	380	11

Section IV. References:

1. Dolomanov, O.V., Bourhis, L.J., Gildea, R.J, Howard, J.A.K. & Puschmann, H. *J. Appl. Cryst.* **2009**, *42*, 339-341.

2. Sheldrick, G.M. *Acta Cryst*. **2015**, *A71*, 3-8.

3. Sheldrick, G.M. *Acta Cryst*. **2015**, *C71*, 3-8.

4. MOPAC2016, James J. P. Stewart, *Stewart Computational Chemistry*, Colorado Springs, CO, USA, **2016**, HTTP://OpenMOPAC.net.

5. Stewart J. J. P., Optimization of parameters for semiempirical methods VI: more modifications to the NDDO approximations and re-optimization of parameters. *J. Mol. Modeling.* **2013**, *19*, 1-32.

6. Dong, M.; Liu, X.; Zhang, Z.-Y.; Yu, C.; Huo, B.; Li, C. Synthesis of a large-cavity carbazole macrocycle for size-dependent recognition. *Chem. Commun.* **2022,** *58*, 2319-2322.
